# Accelerated 2D multi-slice first-pass contrast-enhanced myocardial perfusion using through-time radial GRAPPA

**DOI:** 10.1186/1532-429X-16-S1-P378

**Published:** 2014-01-16

**Authors:** Jesse I Hamilton, Kestutis Barkauskas, Nicole Seiberlich

**Affiliations:** 1 Biomedical Engineering, Case Western Reserve University, Cleveland, Ohio, USA

## Background

Non-Cartesian parallel imaging is a promising approach for reducing the scan time in multi-slice first-pass myocardial perfusion imaging, allowing increasing volumetric coverage in comparison with standard techniques. Through-time radial GRAPPA has previously been demonstrated for real-time functional cardiac imaging [Seiberlich, et al. MRM 2011 Feb;65(2):492-505]. In this work, the through-time radial GRAPPA technique is applied for the acquisition of fifteen slices per heartbeat during a contrast-enhanced myocardial perfusion examination.

## Methods

Three healthy volunteers underwent first-pass myocardial perfusion imaging on a 3T Skyra scanner with a 34-channel array. For calibration of through-time radial GRAPPA, 26 frames of fully-sampled data were collected for 15 slices (scan time 2.7 minutes) without ECG gating or breath holds using the following parameters: radial FLASH sequence, 128×128 grid, TR = 2.94 ms, TE = 1.47 ms, FOV = 300 mm2, spatial resolution = 2.3 mm2, 8 mm slice thickness. After injection of contrast agent (Optimark, 0.1 mmol/kg, 3 mL/s), undersampled radial data with ECG gating were collected for 60 cardiac phases. For the accelerated scans, 12 projections per slice were collected (R = 16.8 with respect to the Nyquist limit), and volunteers were instructed to hold their breath for as long as possible. A non-selective saturation recovery pulse was played after each cardiac trigger, and data for all slices were collected within each cardiac phase after a set trigger delay. All other parameters were identical to the calibration scans. The through-time radial GRAPPA reconstruction was performed using 26 time repetitions and an 8×4 k-space segment, and data were gridded using an open-source NUFFT implementation [Fessler JA. JMRI 2007 Oct;188(2):191-195].

## Results

**Figure 1:**
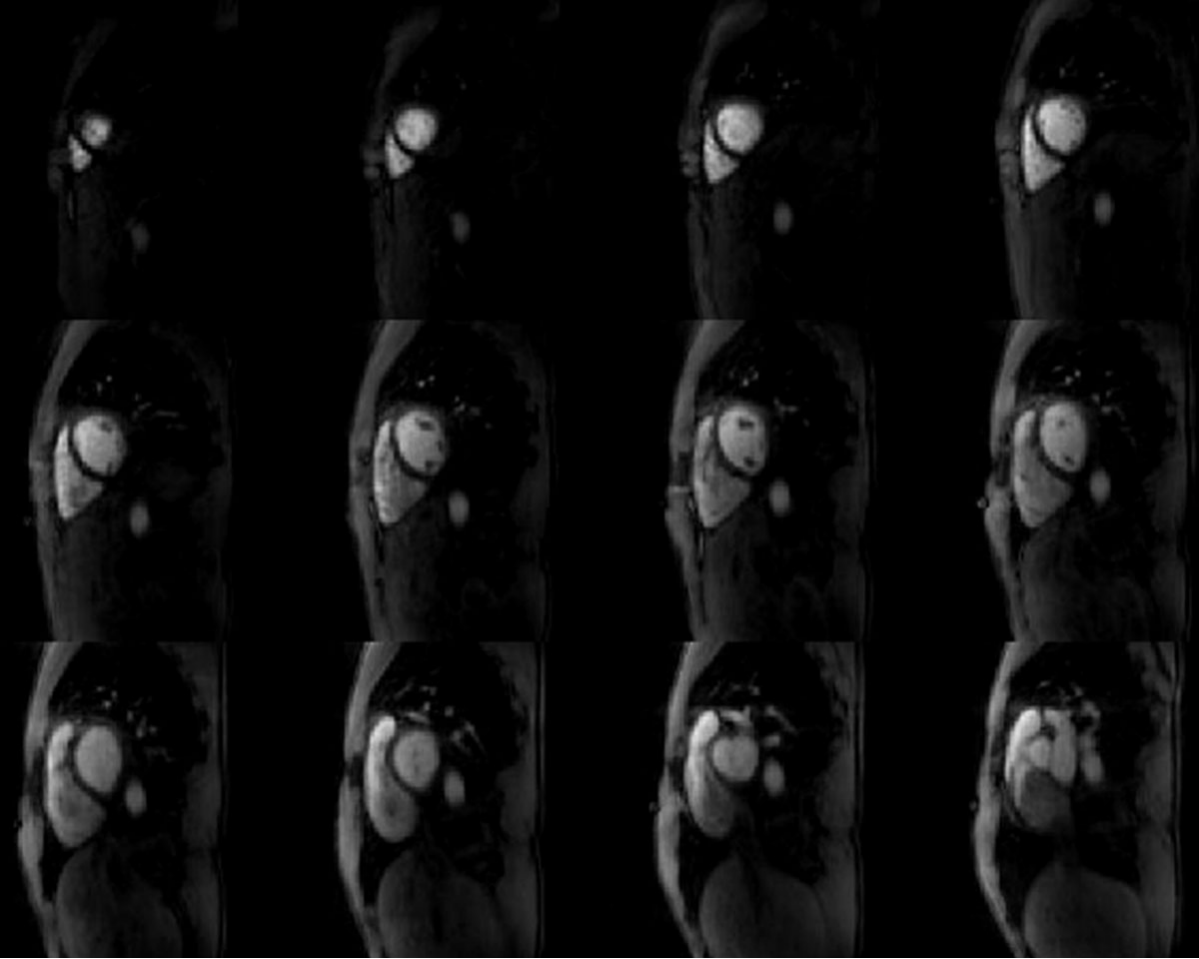
**Representative images reconstructed with through-time radial GRAPPA from twelve out of fifteen slices during peak enhancement**.

**Figure 2:**
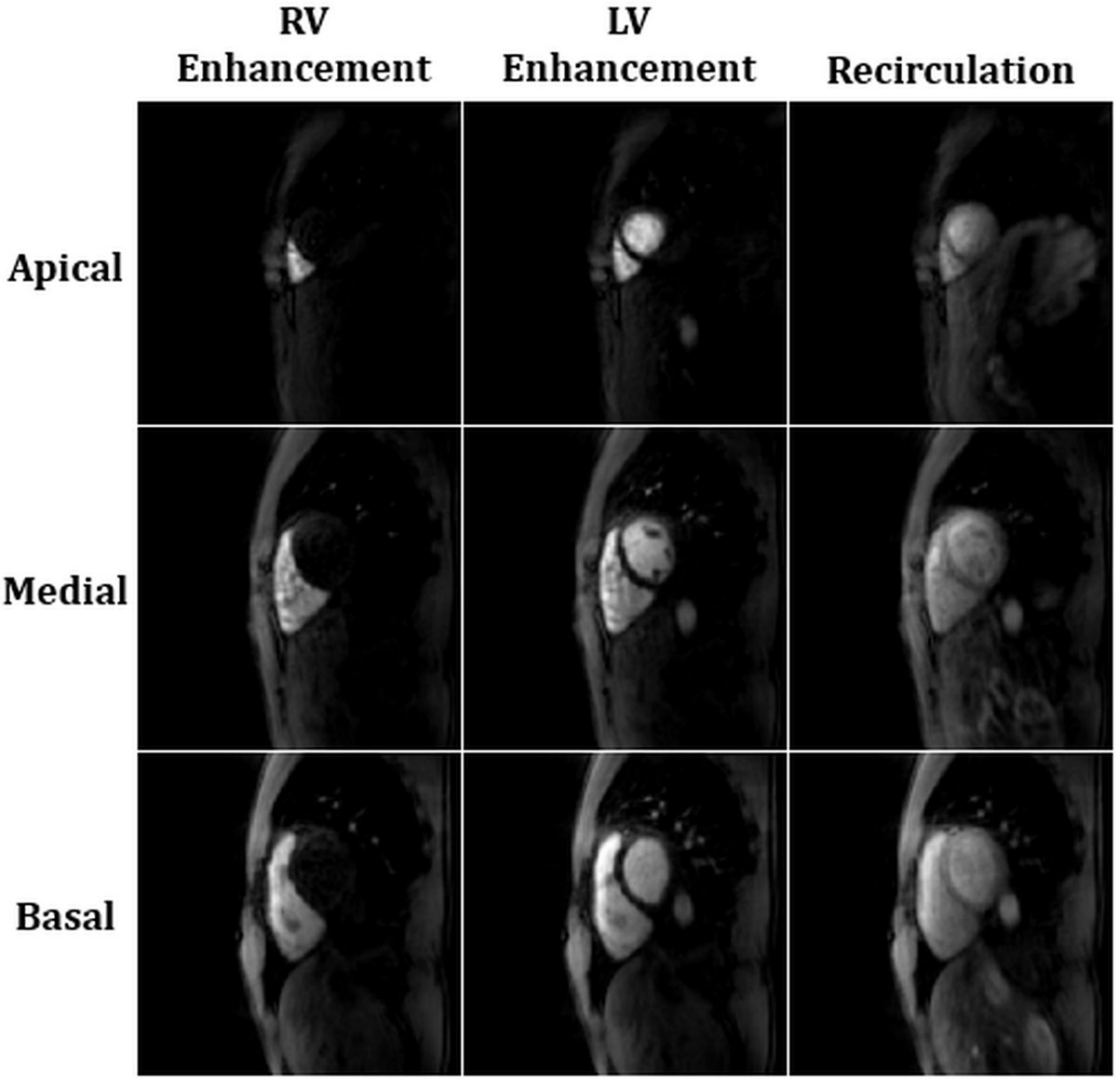
**Images from three slices reconstructed with through-time radial GRAPPA, showing enhancement of the right ventricle, peak left ventricular enhancement, and recirculation**.

Representative images during peak enhancement from twelve out of fifteen slices are shown in Figure 1. Despite the large acceleration factor, no residual streak artifacts are observed. Anatomical structures such as the myocardial wall and papillary muscles can clearly be identified. Due to the short temporal footprint, fifteen slices could be imaged in 573 ms every cardiac cycle. Figure 2 shows images for three slices (apical, medial, and basal) during three key time points: right ventricular enhancement, peak enhancement, and recirculation.

## Conclusions

It has been demonstrated that through-time radial GRAPPA can be used for 2D multi-slice first-pass contrast enhanced myocardial perfusion. By using a high acceleration factor, up to fifteen slices can be imaged within each heartbeat.

## Funding

NIH/NIBIB R00EB011527 and Siemens Medical Solutions.

